# Evaluation of human antibodies from vaccinated volunteers for protection against *Yersinia pestis* infection

**DOI:** 10.1128/spectrum.01054-24

**Published:** 2024-08-27

**Authors:** Li Zhang, Binyang Zheng, Jing Lu, Haisheng Wu, Hailian Wu, Qi Zhang, Lei Jiao, Hongxing Pan, Jianfang Zhou

**Affiliations:** 1 National Key Laboratory of Intelligent Tracking and Forecasting for Infectious Diseases (NITFID), National Institute for Viral Disease Control and Prevention, Chinese Center for Disease Control and Prevention, Beijing, China; 2 Jiangsu Provincial Center for Disease Control and Prevention, Nanjing, China; 3 Qinghai Institute for Endemic Disease Control and Prevention, Xining, China; 4 Lanzhou Institute of Biological Products Co., Ltd., State Key Laboratory of Novel Vaccines for Emerging Infectious Diseases, Lanzhou, China; Institut National de Santé Publique du Québec, Québec, Canada

**Keywords:** *Yersinia pesti*, phage display, fraction 1 capsular antigen, human monoclonal antibody, animal protection

## Abstract

*Yersinia pestis* has a broad host range and has caused lethal bubonic and pneumonic plague in humans. With the emergence of multiple resistant strains and the potential for biothreat use, there is an urgent need for new therapeutic strategies that can protect populations from natural or deliberate infection. Targeting F1 has been proven to be the main strategy for developing vaccines and therapeutic antibodies, but data on anti-F1 antibodies, especially in humans, are scarce. To date, three human anti-F1 monoclonal antibodies (m252, αF1Ig2, and αF1Ig8) from naive populations have been reported. Here, we constructed an antibody library from vaccinees immunized with the plague subunit vaccine IIa by phage display. The genetic basis, epitopes, and biological functions of the obtained mAbs were assessed and evaluated in plague-challenged mice. Three human mAbs, namely, F3, F19, and F23, were identified. Their biolayer responses were 0.4, 0.6, and 0.6 nm, respectively. The dissociation constants (K_D_) of the F1 antigen were 1 pM, 0.165 nM, and 1 pM, respectively. Although derived from distinct Ab lineages, that is, VH3-30-D3-10-JH4 (F3&F23) and VH3-43-D6-19-JH4 (F19), these mAbs share similar binding sites in F1 with some overlap with αF1Ig8 but are distinct from αF1Ig2. Each of them provided a significant protective effect for Balb/c mice against a 100 median lethal dose (MLD) challenge of a virulent *Y. pestis* strain when administered at a dose of 100 µg. No synergistic or antagonistic effects were observed among them. These mAbs are novel and excellent candidates for further drug development and use in clinical practice.

**IMPORTANCE:**

In this study, we identified three human monoclonal antibodies with a high affinity to F1 protein of *Yersinia pestis*. We discovered that they have relatively lower somatic hypermutations compared with antibodies, m252, αF1Ig2, and αF1Ig8, derived from the naive library reported previously. We also observed that these mAbs share similar binding sites in F1 with some overlapping with αF1Ig8 but distinct from that of αF1Ig2. Furthermore, each of them could provide complete protection for mice against a lethal dose of *Yersinia pestis* challenge. Our data provided new insights into the anti-F1 Ab repertories and their associated epitopes during vaccination in humans. The findings support the additional novel protective human anti-F1Abs for potential therapeutics against plaque.

## INTRODUCTION

*Yersinia pestis* (*Y. pestis*), a nonmotile gram-negative bacterium, is the causative agent of plague, a disease that has resulted in three pandemics and over 150 million deaths worldwide (1–3). *Y. pestis* is highly pathogenic to mammals, including humans, and can cause severe and often fatal infections, with a mortality rate of up to 100%. In the 21st century, cases of *Y. pestis* infection and plague have been reported in Africa, Asia, and South America (4). Currently, Uganda (5), China (6), the Democratic Republic of the Congo (7), and Madagascar (8, 9) are considered focal points for plague outbreaks globally. *Y. pestis* has raised international public health concerns due to its ability to be transmitted naturally and its potential as a bioterrorist weapon (10).

Integrated interventions, including antibiotics, vaccines, therapeutic antibodies(Abs), and rodent and flea elimination, are essential for the control of plague. Antibiotics are effective in treating plague at an early stage, as *Y. pestis* is inherently susceptible to all antibiotics. However, strains with transferable plasmids containing multiple antibiotic resistance genes (ARGs) have been identified in Madagascar (11, 12). Vaccination is another method for preventing plague, and options such as attenuated vaccines (13), killed whole-cell vaccines, and subunit vaccines (14, 15) show promise in protecting *Y. pestis* (16). The live attenuated vaccine EV76 and the relevant derivatives have been widely applied to millions of people without severe complications since the 20th century (17, 18). It has been proven that this vaccine protects against both bubonic and pneumonic plague, and it is still in use mainly in Kazakhstan (19) and Russia (20). However, studies demonstrated that mice (21) and rhesus macaques (22) immunized with EV76 have a higher titer to fraction 1 capsular antigen (F1) but no detectable level of antibodies against low-calcium-response V antigen (LcrV). Recently, the development of vaccines has focused primarily on the F1 and LcrV (23). Most individuals could produce systemic Abs to both F1 and LcrV after being primed by a divalent subunit vaccine of F1 + rLcrV *via intramuscular injection (i*.*m*.) (24).

Many lines of investigations support the protective effects of poly- and monoclonal- Abs (mAbs) targeting T3SS (LcrV, YopB, and YopD), Yops, and F1 against plague. In particular, F1, which is an antiphagocytic capsular antigen, serves as the primary protective antigen and can be secreted in the host during infection (25). Additionally, F1 serves as a target for the preparation of therapeutic antibodies, as mAbs targeting F1 have been shown to protect mice against fatal pneumonic and bubonic plague caused by wild-type F1 + microbes (26). Furthermore, anti-F1 and anti-LcrV Abs might be synergistic. Intratracheal delivery of aerosolized anti-LcrV and anti-F1 could protect mice challenged with pneumonic plague (27). Both anti-F1 and anti-LcrV mAs could not only increase bacterial uptake by macrophages but also downregulate specific cytokine response during infection that is also associated with *in vivo* protection (28). Of note, the direct anti-bacterial effects via mAbs and their immune-regulatory effects in host function independently (29). Fully human polyclonal Abs derived from tanschromosomic bovines vaccinated with the F1 + LcrV vaccine could increase bacterial opsonization *in vitro* and protect against pneumonic plague *in vivo* (30). Till now, F1-specific mAbs are usually derived from either immunized animal or human-naive antibody libraries (28, 31, 32). In the context of immunotherapy in humans, the use of mouse mAbs was found to be linked with the development of a human anti-mouse antibody (HAMA) response and severe adverse reactions following initial dosing. Nevertheless, the data on anti-F1 generated by humans acquired naturally or via vaccination are poor, and only three human anti-F1 mAbs, that is, m252 (32), αF1Ig2, and αF1Ig8 (33), from naive population have been reported previously.

In this study, we presented three human antibodies from the antibody libraries of F1 + LcrV-vaccinated adults. The genetic basis, epitopes, and biological functions of the obtained mAbs were assessed. We also evaluated them in plague-challenged mice. We found that they exhibited protections against the *Y. pestis* challenge. Our data provided new insights into the anti-F1 Ab repertories and their associated epitopes during vaccination in humans. Consequently, the findings on human antibodies provide an alternative in diagnostics, therapeutics, and fundamental research on *Y. pestis*.

## RESULTS

### PBMC isolation and plasma evaluation

We collected plasma and peripheral blood mononuclear cells (PBMCs) 28 days after the first vaccination from five candidates in phase II clinical trial of a recombinant subunit plague vaccine (F1 + rV) (34). Serum F1 antibody titers were evaluated by indirect enzyme-linked immunosorbent assay (ELISA). The five donors had significantly greater anti-F1Ab titers (Fig. S1) than the preimmunization serum donors. The geometric mean antibody titers (GMTs) against F1 were greater than 1,000. These results indicated that all five volunteers had a good humoral immune response against the F1 antigen. Approximately 1 × 10^7^ PBMCs per sample were obtained.

### Generation and screening of antibodies against *Y. pestis*

The mixed complementary DNA (cDNA) from PBMCs was used to amplify variable fragments of VH and VL chains (350 bp each). The single-chain antibody fragments (ScFvs) were constructed by overlapping PCR and were ~700 bp in length. After sequencing, an antibody library with a complexity of 7.0 × 10^7^ independent clones and 100% diversity was established. In each round of panning, 5 × 10^11^ plaque-forming units (PFU)/mL phage plasmids were added. After three successive rounds of panning, approximately 100-fold enrichment was achieved. These results indicated that the enrichment ratios were normal. Enrichment was defined as the output/input ratio in any panning round compared with the same ratio in the first panning round (Table S1). Finally, 96 clones were picked and screened by phage-ELISA, and 32 positive monoclonal antibodies (mAbs) were identified.

### Sequence alignment analysis for the generated antibodies

**Fig 1:**
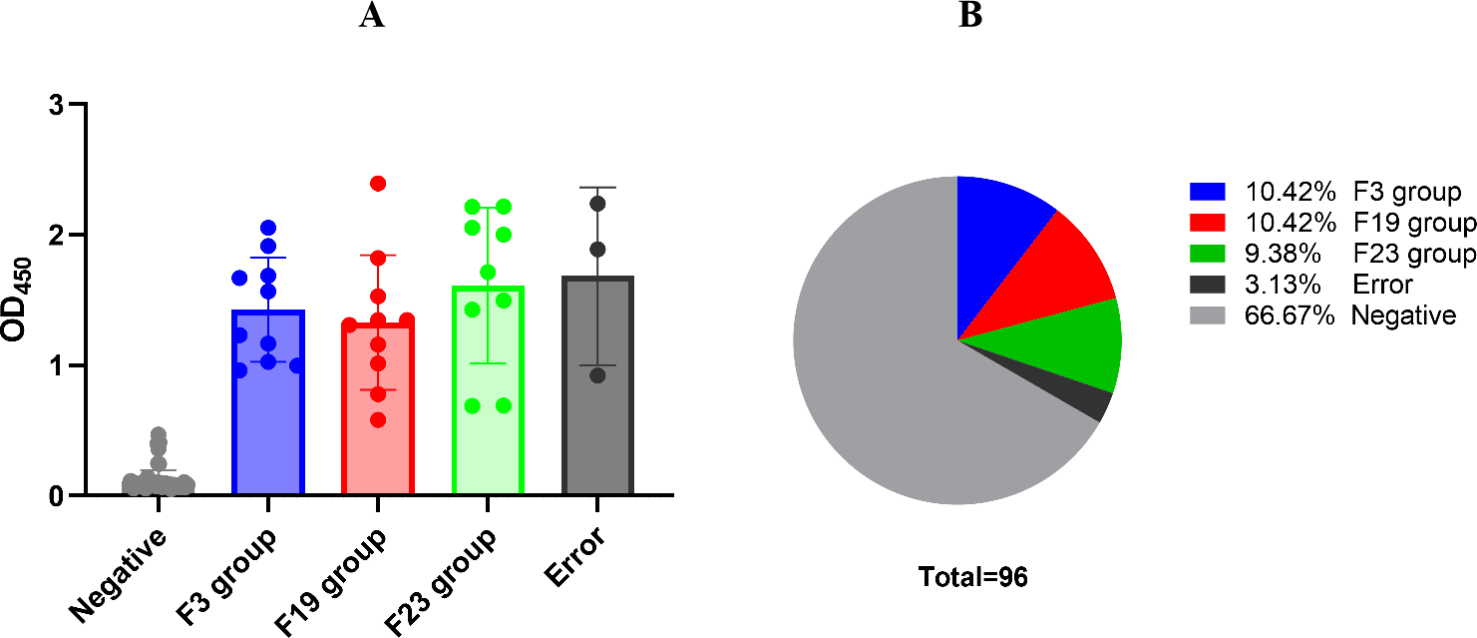
Panning results of phage library by F1 antigen. (A) Ninety-six randomly picked colonies were screened by ELISA to evaluate binding activity to F1 antigen. The data are expressed as the absorbance at 450 nm. Thirty-two phage antibodies binding to F1 were sequenced. Variable gene sequences of heavy and light chains were run through the blast and aligned with homologous sequences of the IMGT (www.imgt.org/) database. Three unique antibodies (F3, F19, and F23) were identified, and the abundance of each group was depicted in the pie chart (B). The error denotes the arrangement or unproductive for the sequences.

All F1-binding clones were sequenced, and their closest germline gene matches were identified using the IMGT/V-Quest tool. Paired sequences were aligned by ClustalW methods in MEGA software (version 6.0). FastTree 2.3 was used to construct a phylogenetic tree (Fig. S2). In total, 29 productive antibody sequences were obtained from 32 positive clones. However, three clones were incomplete or had pseudogenes. Based on the diversity of variable genes determined by sequencing alignments and the distance in the phylogenetic tree, as well as the optical density (OD) values for the detection of F1 binding, three antibody groups, F3, F19, and F23, were selected as candidates for further assays (Fig. 1A). The percentages of the VH lineage composed of F3 (*n* = 10), F19 (*n* = 10), and F23 (*n* = 9) were 10.42%, 10.42%, and 9.38%, respectively (Fig. 1B). The VH gene families of F3, F19, and F23 were IGHV3-30*18, IGHV3-43*02, and IGHV3-30*04, respectively. For the light chain, the VK gene families were IGKV1-39*01, IGKV1-39*01, and IGKV4-1*03. In addition, the D and J gene families, as well as the somatic mutation rates, are listed in Table 1. Furthermore, the full lengths of the variable region amino acids F3, F19, F23, m252, α F1Ig2, α F1Ig8, and JS06 (an isotype control antibody) were aligned by using the web servers ESPrit3.0 (http://espript.ibcp.fr/ESPript/ESPript/). The complementarity-determining regions (CDRs) of F3, F19, and F23 did not show any similarities with the documented amino acid sequences of human anti-F1 mAbs and JS06 (Fig. S2 and S3).

**TABLE 1:** The gene characteristics of F3, F19, and F23^*a*^

Ab clone	V gene	Somatic mutation rate in V gene (% nucleotide)	D gene	J gene	Somatic mutation rate in J gene (% nucleotide)	Amino acid no. of CDR
F3	IGHV3-30*18	3.82	IGHD3-10*0	IGHJ4*02	10.64	8.8.9
	IGKV1-39*01	0.36	-	IGKJ1*01	0.00	8.3.8
F19	IGHV3-43*02	3.12	IGHD1-1*01	IGHJ4*02	8.51	8.8.11
	IGKV1-39*01	1.08	-	IGKJ1*01	2.78	8.3.8
F23	IGHV3-30*04	2.43	IGHD3-10*01	IGHJ4*02	12.77	8.8.18
	IGKV4-1*03	4.38	-	IGKJ2*01	8.11	12.3.9

^*a*^ The V, D, and J sequences were compared by using the IMGT database. ‘-’ denotes the gene without the segment.

### Characterizing the binding profile of candidate antibodies

**Fig 2:**
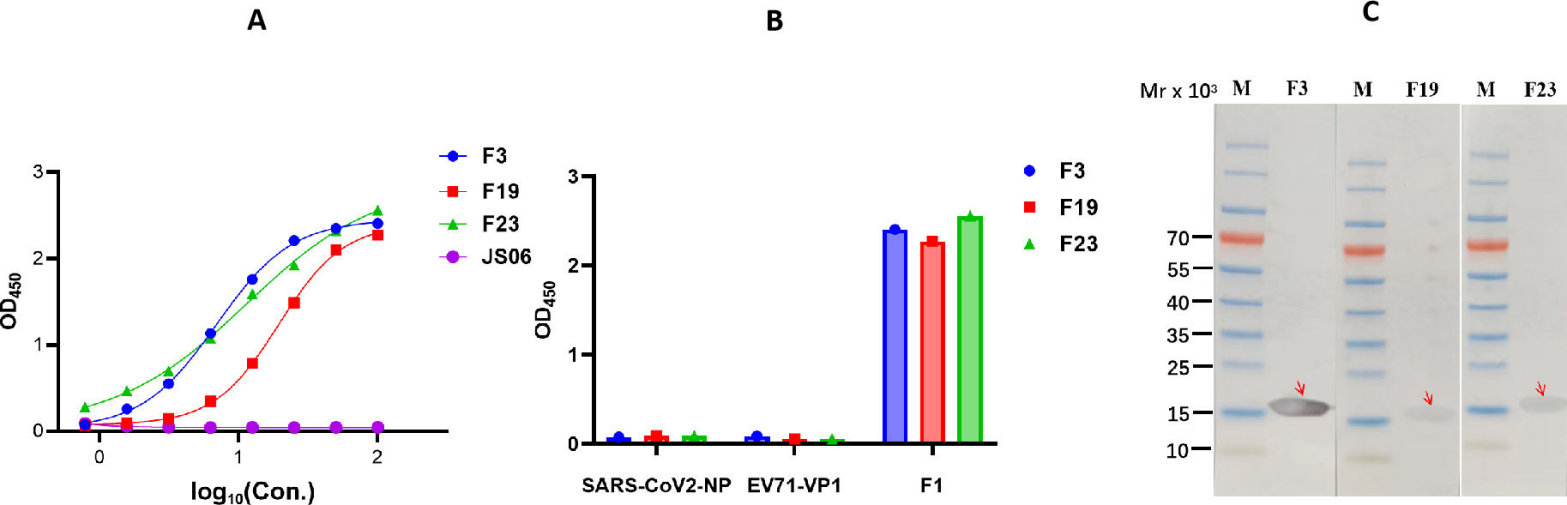
Binding characteristics of three mAbs to F1 antigen. (A) ELISA binding curves of F3, F19, and F23 against F1 antigen of *Y. pestis*. The horizontal axis shows different concentrations of F1-specific mAbs. “JS06” denotes the irrelevant control antibody. The data were expressed as the average of triplicate measurements of absorbance at 450 nm. Their EC50 values were 6.92 ng/mL, 19.4 ng/mL, and 11.36 ng/mL, respectively. (B) Cross-reactivity against two unrelated antigens, SARS-CoV-2 N and EV71-VP1 were evaluated by ELISA. F1 antigen was used as a positive control. (C) Western blot analysis of antibodies using purified F1. F1 antigen was electrophoresed and transferred to membranes, and then, the three mAbsdetected it. After incubation with the secondary antibody, Diaminobenzidine (DAB) was used to detect the immunoreactive bands.

The full-length human IgG1 antibody was purified and prepared at 1 mg/mL for ongoing use. Both indirect ELISA and western blot (WB) were performed to determine the binding specificity between the three mAbs and F1. For the indirect ELISA, coated F1 was recognized by three mAbs. The binding curves of F3, F19, and F23 against the F1 antigen are shown in Fig. 2A. Their half-maximal effective concentration (EC 50) values were 6.92 ng/mL, 19.4 ng/mL, and 11.36 ng/mL, respectively. However, none of these mAbs cross-reactive with irrelevant antigens (SARS-CoV-2 NPs and EV71-VP1), as shown in Fig. 2B. All antibodies were further evaluated by WB analysis with F1. As shown in Fig. 2C, the F3 antibody could bind to F1, which displayed a band with a size of 15.5 kDa, whereas a relatively weak band was observed for F19 and F23, suggesting relatively weak binding affinity.

### F3, F19, and F23 exhibit high affinities for F1

**Fig 3:**
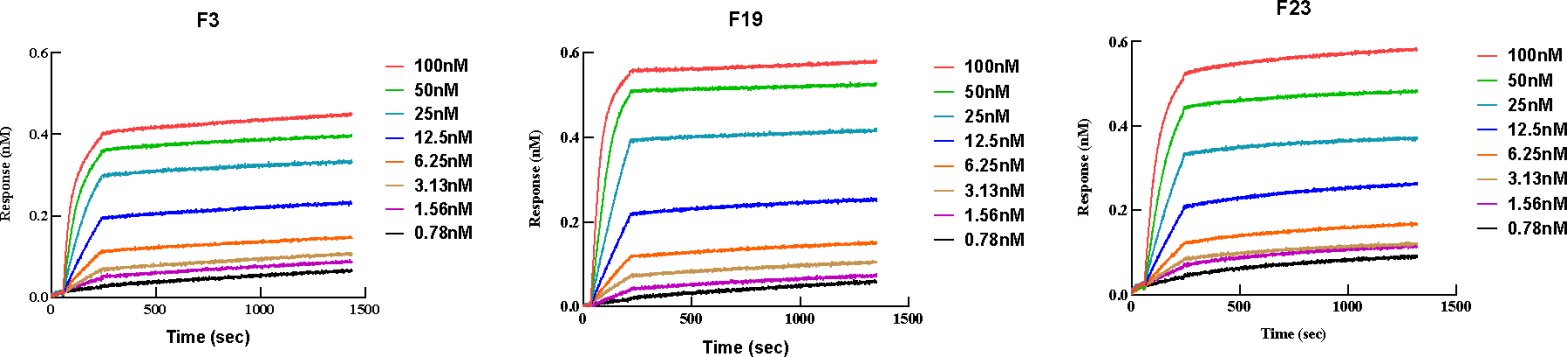
The binding affinity and kinetic of three mAbs against F1 antigen. Binding kinetics measurements were performed using Bio-Layer Interferometry on ForteBio Red Octet 96 instrument. The K_D_ values of F3 and F23 antibodies were both less than 1 pM, whereas the K_D_ value of F19 was 0.165 nM.

A biolayer interferometry (BLI) assay was performed to evaluate the binding specificity and affinity between the antibodies and purified F1. The dissociation constant (K_D_) values of the F3 and F23 antibodies were both approximately 1 pM, whereas the K_D_ value of F19 was 0.165 nM. All kinetic curves of the three antibodies were recorded and are depicted in Fig. 3. Our results showed that all three candidate antibodies exhibited a tight binding affinity for F1, but the binding modes differed among the three mAbs. The F3 antibody, which exhibited a clear band in the WB image, bound less to F1, and the maximum thickness of the biolayer was 0.4 nm. The thicknesses of F19 and F23 were both approximately 0.6 nm. From these results, we concluded that F19 and F23 could capture more antigen than the F3 antibody in the BLI assay.

### Antigen epitope recognized by F3, F19, and F23

**Fig 4:**
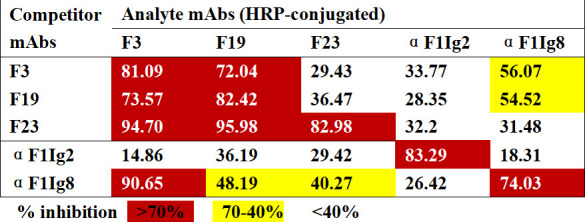
Competition map of mAbs binding by ELISA. Human mAbs (in red) and mAbs reported previously (in blue) were HRP-conjugated and used for competition binding F1 antigen with mAbs listed in the leftmost column. Percent inhibition of analyte mAbs binding by competitor mAbs is indicated by color as shown in the color key. Competition binding curves are shown in Fig. S4.

In the peptide-ELISA, 22 peptides covering the full length of F1 were synthesized (Table S3) and detected. However, all the mAbs, including α F1Ig2 and α F1Ig8, did not bind to any peptide (data not shown). To determine whether mAbs recognize similar or different epitopes, binding competition assays were performed via ELISA. The percent inhibition of analyte (horseradish peroxidase (HRP)-conjugated) mAb binding by competitor (unlabeled) mAbs is shown in Fig. 4, and competition curves are shown in Fig. S4. Based on the data, F3-HRP binding to the F1 antigen was almost completely blocked by F19 and F23, with percent inhibition greater than 70%. These results suggested that these three human mAbs had similar binding sites. The same result was also obtained with the HRP-conjugated F19 antibody. However, HRP-conjugated F23 strongly inhibited unlabeled F23 itself, but low competition was found between F3 and F19. In the control group, α F1Ig2 did not significantly compete with α F1Ig8 or the other three human mAbs. However, α F1Ig8 had a moderate inhibitory effect (ranging from 40%–90%) on F3, F19, and F23. This finding suggested that the epitopes of α F1Ig8 had a certain degree of overlap with F3, F19, and F23. Therefore, the combined data demonstrated that the binding sites of the three human mAbs partly overlapped with those of α F1Ig8 but did not completely overlap with those of α F1Ig2.

### F3, F19, and F23 protect mice against lethal plague

Mice were given purified mAb by i.p. injection on the day before the challenge with 100 MLD of the *Y. pestis* 141 strain. All mice, including the passively immunized and nonimmunized mice, were monitored for health status and survival time after the challenge. The results of the pre-experiment showed that all mice were 100% protected by 500 µg and 100 µg of each antibody and antibody mixture. There were no deaths recorded within 20 days after the challenge with 100 MLD of the *Y. pestis* 141 strain. In parallel, the average survival time for the 100 MLD-treated group (nonimmune antibody) was 4.875 days (Table S4).

**Fig 5:**
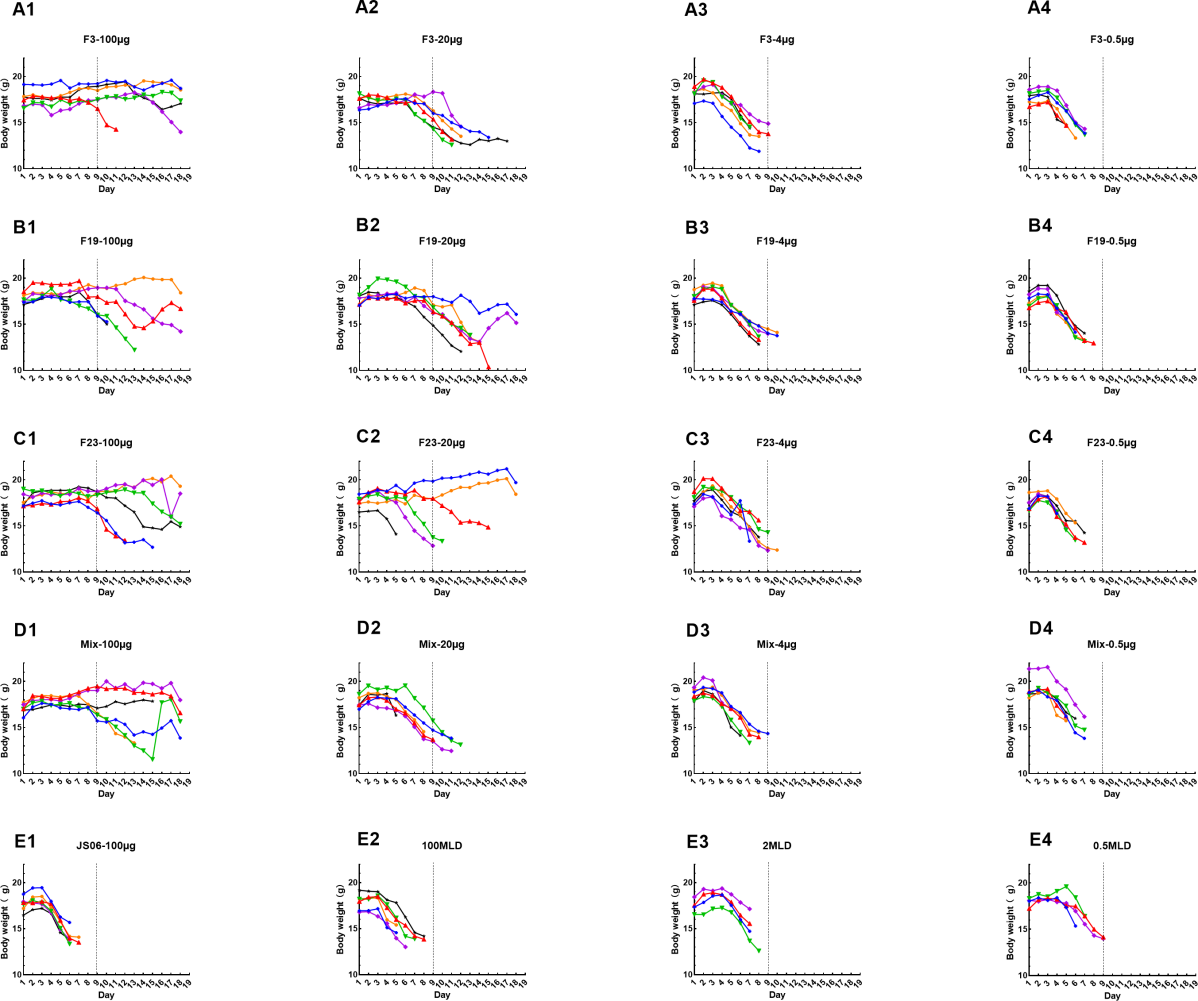
Body weight changes of mice in *in vivo* animal experiment. A1­-A4. Body weight change curves of mice immunized by serial doses(100, 20, 4, and 0.5 µg）of F3 mAb. B1-B4. Body weight change curves of F19 group’s mice. C1-C4. Body weight change curves of F23 group’s mice. D1-D4. Body weight change curves of mice immunized by serial dose mix mAbs (F3, F19, and F23 were mixed in equal amounts). E1. Boy weight changes of JS06 (anti-SARS-CoV-2 N human antibody) as a same-isotype irrelevant control in 100 µg; E2­E4. Body weight changes of mice were challenged only by sequential doses of *Y. pestis* (100, 2, and 0.5 MLD). Mice were randomly assigned to A1-E4 group (*n* = 8 per group); each color represents an individual animal trajectory in each group.

In the formal experiment, F3, F19, and F23 as well as the mixture group (F3, F19, and F23 were mixed in equal amounts) were evaluated systematically. Each individual mouse’s trajectory was recorded and depicted as follows. The actual body weight changes of each mouse are shown in Fig. 5. Based on the data, we can get the following results. First, in the 100 µg group of each experiment, about 71% of the mice (7/24) survived at the end of the experiment. However, no obvious increase in body weight was found in any of the surviving mice, indicating that the *Y. pestis* infection in mice is generally severe and pathogenic. The body weight of the 20 µg-treated group decreased by nearly 20%, which was greater than that of the 100 µg group (a decrease of approximately 5%). However, the decreases in the 4 µg, 0.5 µg, irrelevant antibody, and MLD (100 MLD, 2 MLD, and 0.5 MLD) groups were all greater than 20%. The body weight of the mixture-treated group was indistinguishable from that of the single-tested antibody group. No synergistic effects were observed among F3, F19, and F23.

**Fig 6:**
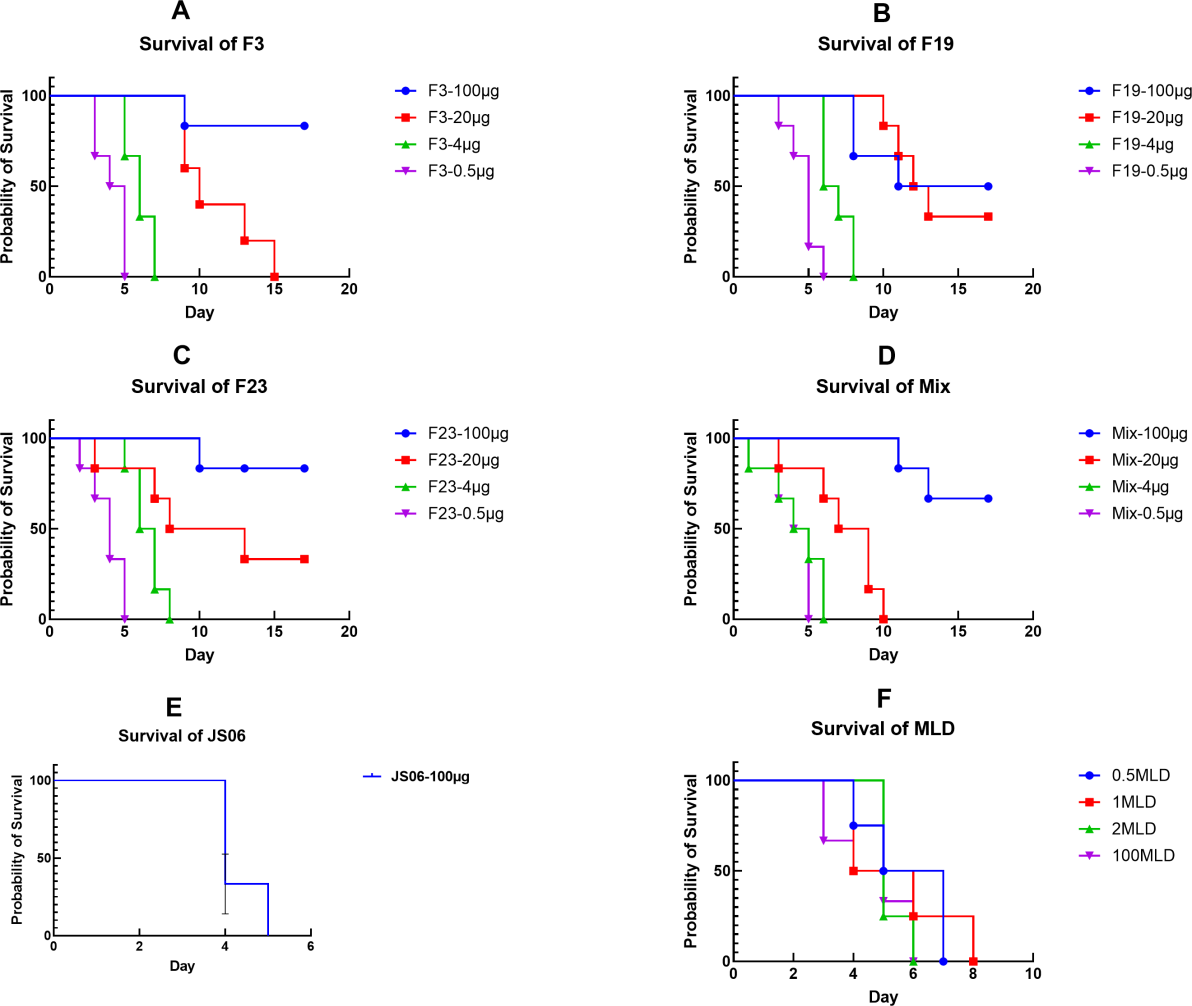
Survival curve of the mice passively pre-immunized by three anti-F1 mAbs, the mixture, and the irrelevant antibody, an anti-N mAb, then challenged by 100 MLD *Y. pestis*. (A–­D) Survival curves of F3, F19, F23, and antibody mixture at 100, 20, 4, and 0.5 µg for each mouse; (E) The survival curve of JS06 (anti-SARS-CoV-2 N human antibody) as a negative control in 100 µg. (F) Survival curves of mice challenged by a sequential dose of *Y. pestis* 141 strain (100, 2, 1, and 0.5 MLD) for bacterial back-titration.

Figure 6 shows strong correlations between the survival curve and body weight, and mice died if their body weight loss was greater than 20%. One hundred micrograms of F3, F19, and F23 antibodies were all protective against *Y. pestis*, but 100 µg of JS06 (irrelevant antibody) had no protective effect. The 20 µg group exhibited limited protection, whereas the 4 µg and 0.5 µg groups did not exhibit any protection. No better results were found for the mixture group than for the single mAb group at any dose. We found no detectable synergistic or antagonistic effects of these three mAbs. In the MLD groups, the bacterial back-titration had no dose effect, suggesting that a higher germ dose was used in the experiment. All animals in the 0.5MLD group died within 9 days, whereas no deaths occurred in the 100 µg groups of F3, F19, F23, or the mixture group.

To verify that the deaths were caused by *Y. pestis* infection, bacterial culture and bacteriophage typing were performed. In Congo red agar, non-pigmented colonies were formed, and *Y. pestis*-specific lytic bacteriophages could lyse these colonies.

## DISCUSSION

In this study, we sampled the PBMCs of volunteers 1-month post-immunization with a plague subunit vaccine in a phase II trial and cloned the corresponding antibody genes. Using phage display, we identified three human anti-F1 mAbs, F3, F19, and F23, from an ScFv library. They exhibited high affinity and specificity for the F1 antigen. Importantly, the mAbs showed apparent protection of mice from lethal plague.

Passive immunization, including the use of convalescent plasma and monoclonal antibodies, is widely used for prophylaxis and treatment of numerous diseases, such as infectious diseases, cancer, and autoimmune disorders. In recent years, monoclonal antibodies have emerged as a prominent area of research in both basic and clinical medicine (35, 36). For example, the ZMapp antibody cocktail was developed for the treatment of Ebola virus disease (37), and broadly neutralizing monoclonal antibodies directed against HA, MEDI8852, were used for anti-flu (38). In the context of bacterial-induced infectious diseases, antibiotics demonstrate a notable bactericidal effect on bacteria; however, they do not confer protection against bacterial toxins. In contrast, the use of neutralizing antibodies targeting bacterial toxins is anticipated to serve as a valuable component of combination therapy for infectious diseases. Research has shown that neutralizing antibodies against bacterial toxins, specifically those produced by *Staphylococcus aureus* (39, 40), *Bacillus anthracis* (41), and *Corynebacterium diphtheriae* (42), have been identified and validated for potential clinical application.

In the history of plague treatment, plasma was utilized more than a century ago, with subsequent advancements leading to the development and recommendation of several mAbs for plague treatment. Notably, mAbs targeting LcrV and the F1 capsule, such as F1-04-A-G1 (26) and F2H5 (31), have demonstrated efficacy in providing complete protection against *Y. pestis* in murine models. Additionally, MAb7.3 (43), which binds to a linear epitope in LcrV (residues 135–275), represents the first reported LcrV-specific monoclonal antibody capable of protecting mice against a fully virulent strain of *Y. pestis*. Furthermore, MAb7.3 has been shown to exhibit a synergistic effect when used in conjunction with F1-04-A-G1 (44). However, it is crucial to note that the F1-specific human antibody m252, obtained from a naive library, exhibited only limited protection against *Y. pestis* infection.

In contrast, two LcrV-specific antibodies, m253 and m254, did not demonstrate any protective effects. Moreover, a synergistic effect was observed when the three antibodies were combined (32). Additionally, αF1Ig2 and αF1Ig8, which were also isolated from a naive library, may have potential applications in the diagnosis and immunotherapy of *Y. pestis* infection (33). We compared the properties of our mAbs with those of documented human anti-F1 mAbs. Our mAbs possess a very high affinity for the F1 antigen, and the K_D_ of F3 and F23 is 1 pM, comparable with that of the most potent published Abs, αF1Ig8 and αF1Ig2, which are approximately 0.08 and 0.23 nM, respectively, as reported (33).

In peptide ELISA, a total of 22 peptides were synthesized by spanning the full length of F1. Fortunately, the P21(SKGGKLAAGKYTDAVTV) in Table S3 was almost the same as the P2 peptide (FFVRSIGSKGGKLAAGKYTDAVTV) (45) and No. 7 peptide (46) in the previous studies. P2 and No. 7 peptide were exposed on the surface of F1 molecule and served as the immunodominant sequence of F1. In this study, we found that F3, F19, and F23 did not bind to any peptide including P21, which is on the surface of F1. Based on these data, we concluded that the binding sites of F3, F19, and F23 represent a conformational epitope on F1. This finding is also supported by the competition assay showing that the antigen epitopes recognized by the three mAbs overlap with that of the bacterium-binding mAb αF1Ig8.

Furthermore, the Ab sequences of F3, F19, and F23 were completely different from those of m252, αF1Ig2, and αF1Ig8. A relatively lower level of somatic hypermutations (SHMs) was observed in F3, F19, and F23, indicating their germline nature (Table S2). This genetic feature implied that the mAbs were elicited rapidly because of the lower number of SHMs required for affinity maturation. In contrast to other reported anti-F1 human mAbs from native libraries, which are highly divergent from their putative germline precursors, these mAbs require extensive affinity maturation pathways to obtain broader antigen profiles. We further demonstrated that 71% of the mice treated prophylactically at a dose of 100 µg survived a lethal challenge.

However, considering the natural existence of fully virulent F1-negative *Y. pestis* strains (47, 48), F1-based vaccines as well as F1-specific protective mAbs would not provide optimal protection against all strains of *Y. pestis* that cause plague. Against this background, one possible strategic approach could be to search for new immunogenic targets for vaccines and mAbs. Recent studies have shown that vaccination of mice with recombinant T3SS needle structure protein YscF provided protection to mice against subcutaneous injection of the fully virulent and encapsulated *Y. pestis* strain CO92 (49, 50) Our ongoing work will target more candidate antigens.

Collectively, these findings support the novelty of the human Abs detected here and indicate that they are excellent candidates for further drug development and use in clinical practice.

## MATERIALS AND METHODS

### Cells, bacterial strains, and control antibodies

EXPi293F cells purchased from Life Technologies were cultured at 37°C under 8% CO_2_ in EXPI293 expression medium (Life Technologies, USA) supplemented with 1% penicillin/streptomycin (Life Technologies, USA). The F1 antigen derived from the EV strain of *Y. pestis* was obtained from Lanzhou Institute of Biological Products Co., Ltd. The patient-derived human monoclonal antibody JS06 against the nucleocapsid protein (N) of SARS-CoV-2 was prepared by our laboratory (51) and was used as a negative control. Three human antibodies against F1 (namely m252, αF1Ig2, and αF1Ig8) were downloaded from previously published articles (32, 33). The antibody expression plasmids were synthesized and expressed in EXPi293F cells. However, m252 shows a very low yield in production. Hence, we do not include m252 in the subsequent experiments.

### Preparation of blood samples from plague vaccine participants

Five whole blood samples were collected from a phase IIa clinical trial of the subunit plague vaccine (trial registration number: NCT02596308). PBMCs were isolated by using Ficoll-Paque Plus (GE Healthcare, Boston, MS, USA) density gradient media according to the manufacturer’s protocol. Briefly, blood samples were diluted with the same volume of phosphate-buffered saline (PBS) (Gibco, Grandisland, NY, USA). The diluted blood was slowly transferred to Ficoll-Paque in a SepMate-50 tube (Stemcell, Vancouver, Canada). After horizontal centrifugation at room temperature at 800 × *g* for 20 min, the PBMCs were collected and transferred to a new centrifuge tube. Following two washes with PBS, total RNA was extracted from the PBMCs by using an RNeasy Mini Kit (Qiagen, Valencia, CA, USA) following the manufacturer’s instructions.

### Construction of the human antibody phage display library

The ScFvs) were directly cloned and inserted into the phagemid vector pComb3XSS for library construction. The details of the construction, the antibody diversity repertoire, and the other attributes of this phage library have been described elsewhere (52). Briefly, cDNA was synthesized with oligo (dT) primers using a Transcriptor High Fidelity cDNA Synthesis Kit (Roche, Switzerland). Antibody genes encoding the VH1 ~VH7, Vκ1 ~ Vκ6, and Vλ1 ~ Vλ10 families of human immunoglobulin were amplified using 44 primer pairs. The amplified VH and VL genes were gel-purified on agarose, and the ScFv genes were assembled by overlap PCR using the VH and VL fragments as templates. After restriction enzyme digestion by SfiI, the ScFv cassette was ligated with pComb3XSS. These ligation products were electrotransformed into *E. coli* XL1-Blue to form an antibody library. The library diversity was evaluated by sequencing randomly picked clones for each step of library construction, and the complexity of the library was subsequently calculated.

### Panning and screening of a phage antibody library with F1 antigen

The F1 antigen was used for panning the phage antibody library following a previously described procedure (53). In the first round of panning the antibody library, 50 µg of F1 was coated in immune tubes at 4°C overnight. The coating tubes were blocked with 3% milk in PBST at 37°C for 1 h and then washed with 0.1% Tween 20 in PBS (PBST). The phage antibody library was added to the tubes and incubated at 37°C for 2 h. The supernatant was removed, and the tubes were washed with PBST five times. Furthermore, the bound phage particles were eluted with 0.1 M glycine-HCl (pH 2.0) and immediately neutralized with 1 M Tris-HCl (pH 9.0). Twenty micrograms and 10 µg of protein antigen were used for secondary and third panning, respectively, to screen specific binding antibody pools by phage display. After three rounds of panning, 96 single clones were randomly picked and tested by phage ELISA, which was performed essentially as previously described (54).

### ELISA for screening specific binding antibodies

After three rounds of panning, the specificity of individual clones from the enriched phage pool for binding to F1 was evaluated. Briefly, ELISA plates were coated with 100 ng of F1 protein per well and incubated overnight at 4C. Subsequently, the plates were washed three times in PBST and blocked with 1% bovine serum albumin (BSA) in PBST for 1 h at 37°C. Then, the plates were incubated with 50 µL of single clones from the specific binding antibody pool and 50 µL of 1% BSA/PBS at 37°C for 1 h. The wells were washed three times with PBST, and then, 100 µL of a 1:3,000 dilution of horseradish peroxidase (HRP)-conjugated anti-M13 (Sino Biological, 11973-MM05T-H) was added and incubated at 37°C for 1 h. After washing with PBST, tetramethylbenzidine (TMB) was added and incubated at room temperature for 10 min, after which the color change was monitored at 450 nm by adding 50 µL of 2 M H_2_SO_4_ to stop the reaction. The cutoff value was set as the mean of the blank controls plus 2-fold the standard deviation (X + 2 SD). An OD at 450 nm greater than the cutoff value was considered positive. In this way, single phage antibodies against F1 could be screened for further use.

### Sequencing and genetic analyses of antibodies

All positive clones screened by ELISA were sent for Sanger sequencing analysis. We removed reads with incomplete reading frames and non-immunoglobulin sequences and retained only those with significant similarities to the reference IGHV and J genes in the IMGT database. The phylogenetic tree was constructed using MEGA version 6.0. The variable region amino acids were aligned by using the web server ESPrit 3.0 (http://espript.ibcp.fr/ESPript/ESPript/). Based on the distance in the phylogenetic tree and the OD values of indirect ELISA, the candidate antibody plasmids were selected.

### Production of mAbs

To generate recombinant human mAbs, variable region genes of heavy and light chains were cloned and inserted into expression vectors containing human IgG1 heavy chain and Ig kappa or lambda light chain constant regions, respectively. Heavy and light chain plasmids were cotransfected into Expi293F cells by polyethylenimine (PEI) for expression. The Abs were affinity-purified from the culture supernatant using a Protein A column (GE Healthcare) according to the manufacturer’s protocol. Protein concentrations were determined with a Peirce BCA Protein Assay Kit (Thermo Scientific, San Jose, CA, USA).

### Indirect ELISA and western blot analysis

Purified F1 was coated and blocked in ELISA plates, as described above. mAbs were serially diluted from 100 ng/mL to 0.78 ng/mL. All antibodies were added to the plates and incubated at 37°C for 1 h. HRP-conjugated goat anti-human Fc antibody (Sigma) was used as a secondary antibody at 37°C for 1 h. A TMB substrate (Thermo Fisher, Waltham, MA, USA) was used, and the absorbance at 450 nm was measured by a plate reader (Tecan, Baldwin Park, CA, USA). The effective concentration at which 50% of the maximum binding signal (EC50) was in the linear range was calculated using GraphPad Prism 8 (GraphPad Software, San Diego, CA).

For the western blot analysis, the F1 antigen was electrophoresed on an SDS‒polyacrylamide gel and transferred to a polyvinyl fluoride (PVDF) membrane (Bio-Rad). The membranes were blocked with 1% BSA for 1 h at room temperature. The membrane-immobilized antigens were probed with purified human mAbs (20 µg/mL) overnight at 4°C. HRP-conjugated goat anti-human IgG (Sigma, Saint Louis, MO, USA) was used as a secondary antibody. After washing, the membranes were visualized using a diaminobenzidine (DAB) kit (Tiangen, China) according to the manufacturer’s instructions.

### Binding studies by biolayer interferometry (BLI)

Binding kinetics measurements were performed using biolayer interferometry on a ForteBio Red Octet 96 instrument (Sartorius, Inc., Göttingen). Briefly, individual human antibodies were diluted to 10  µg/mL, loaded onto Protein A biosensors (Octet, 185010) for 30 s, and washed to remove any unbound material before conducting measurements in PBST. The immobilized antibodies were incubated with varying concentrations of F1 to capture the kinetic data. All binding data were collected at 30°C. A total of 4–6 concentrations of antigens were used. The baseline and dissociation steps were carried out in a buffer only. The kinetic data were fit to a simple 1:1 binding model to determine the dissociation constant (K_D_) using the association (Kon) and dissociation (Koff) rates. Binding assays were performed in triplicate, and average K_D_ values were calculated.

### Peptide-based ELISA and competitive ELISA

Seventeen amino acid-long (17-mers) peptides overlapping by 10 amino acid residues and spanning the full length of F1 were designed using the Los Alamos National Library web-based software PeptGen (http://www.hiv.lanl.gov/content/sequence/PEPTGEN/peptgen.html) and synthesized (purity >90%; Sangon, Shanghai, China). All the peptides were reconstituted in dimethyl sulfoxide (DMSO) at a concentration of 20 mg/mL and stored at −80°C. Then, the 96-well plate was coated with 50 µL of peptides at 4 µg/mL in PBS overnight. All mAbs were added, and indirect ELISA was performed as described above.

For analysis of the cross-competition between F1-specific mAbs, a competitive ELISA was developed. Purified mAbs were labeled with HRP using an HRP-labeling kit (Sigma Aldrich) following the manufacturer’s instructions. A concentration of HRP-conjugated mAb in the linear range of the titration curve was chosen for competition ELISA. Unlabeled competitor mAbs were serially diluted and mixed with HRP-labeled mAbs. After shaking at room temperature for 20 min, the mixture was added to an F1-coated plate. Following incubation for 30 min at room temperature, the OD values were recorded. The average values of HRP-labeled mAbs alone were considered the binding control. The percentage of HRP-conjugated mAbs bound to F1 was calculated from the OD450 of HRP-Abs with the test Abs divided by the OD450 of HRP-Abs without Abs. The percentage of inhibition was calculated as 100% of the percentage of HRP-conjugated mAbs bound to F1.

### *In vivo* animal experiment

All experiments with live *Y. pestis* were performed in the BSL-3 laboratory at the Qinghai Institute for Endemic Disease Prevention and Control. Six- to 8-week-old female BALB/c mice were purchased from Jiangsu Huachuang Xinnuo Pharmaceutical Technology Co., Ltd., and acclimated for 3 days after arrival. Preexperimentally, the mice were randomly assigned to individual mAb and mAb mixture groups (*n* = 4 per group) or control groups (*n* = 8). To examine the prophylactic efficacy against *Y. pestis*, each mouse received 500 µg or 100 µg of monoclonal antibody by intraperitoneal injection. Twenty-four hours after immunization, all mice were subcutaneously (s.c.) challenged with 100 MLD of a virulent strain*, Y. pestis* 141. All mice were observed for 20 days after the challenge. Once death was confirmed, the mice were dissected, and the heart, liver, spleen, lung, kidney, and lymph node tissues were collected. These organs were plated on Congo red agar and incubated at 28°C for 48 h to observe bacterial growth. The presence of bacteria was confirmed by bacteriophage typing.

In the formal experiment, mAbs were selected based on the results of the preliminary test. Mice were assigned to the mAb group, mixture group, same-isotype irrelevant antibody group, or MLD group (*n* = 24 per group). In each group, mice received four sequential doses of antibodies (100, 20, 4, or 0.5 µg/group) (*n* = 6 per subgroup). The 100 MLD of the *Y. pestis* 141 strain was also used as the challenge dose. Unimmunized mice (*n* = 6) were challenged with a sequential dose of *Y. pestis* (100, 2, 1, and 0.5 MLD) to evaluate the bacterial virulence of *Y. pestis*. The body weights were recorded daily.

## Data Availability

The data are available in the main text or the supplementary information.These three human mAbs（F3,F19 and F23) have been filed in China (patent numbers: CN117683129A,CN117683130A, and CN117683128A).

## SUPPLEMENTAL MATERIAL

Supplemental figures and tables spectrum.01054-24-s0001.docx Fig. S1 to S4; Tables S1 to S4.

ASM does not own the copyrights to Supplemental Material that may be linked to, or accessed through, an article. The authors have granted ASM a non-exclusive, world-wide license to publish the Supplemental Material files. Please contact the corresponding author directly for reuse.
