# Supplementary material for: Evaluation of human antibodies from vaccinated volunteers for protection against Yersinia pestis infection
Source: Microbiol Spectr. 2024 Aug 27;12(10):e01054-24. doi: 10.1128/spectrum.01054-24 (PMC11448073; doi:10.1128/spectrum.01054-24)
Supplement: Supplemental figures and tables — Fig. S1 to S4; Tables S1 to S4. [file spectrum.01054-24-s0001.docx]

Supplementary Information

Evaluation of Human Antibodies from Vaccinated Volunteers for Protection against Yersinia pestis Infection

Zhang et al.,

Number of Supplementary Figures: 4

Number of Supplementary Tables: 4

**FIG S1** **Geometric mean antibody titers against F1 of the plasma from 5 vaccinees.** The horizontal axis shows different time points. “Day 0” means pre-vaccination. “Day 28” means 28 days post-vaccination. The ordinates represent the GMTs for the antisera against F1.


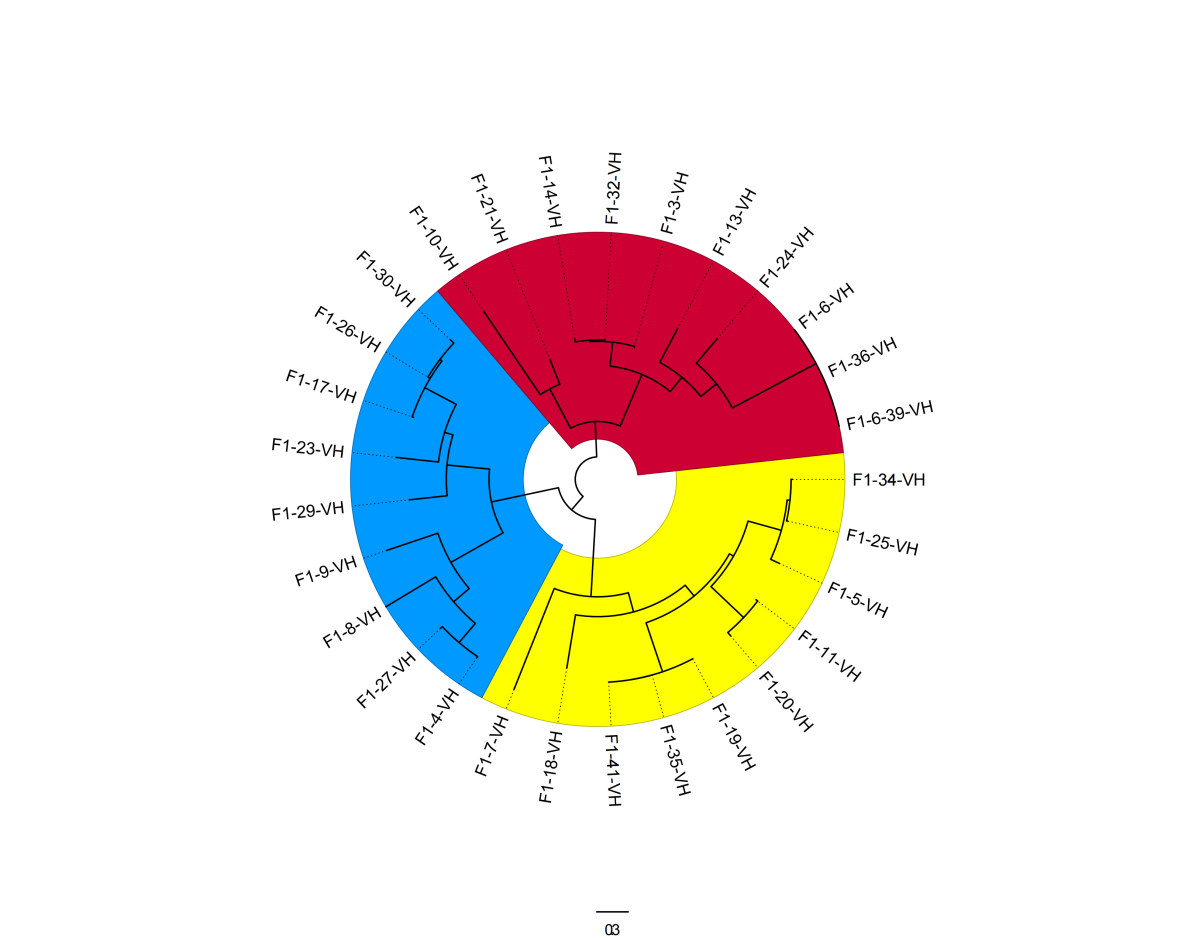


**FIG S2 The** **phylogenetic tree is based on nucleotide sequences of variable regions of heavy chains of selected clones.**

A maximum likelihood phylogenetic tree for the nucleotide sequences of each gene was constructed using the MEGA5.1 program. The tree topology was evaluated by performing 1,000 bootstrap analyses. The bootstrap values ≥ 0.6 are shown at the major nodes of the phylogenetic trees. The VH family of F3, F19, and F23 were highlighted with red, yellow, and blue colors.

A


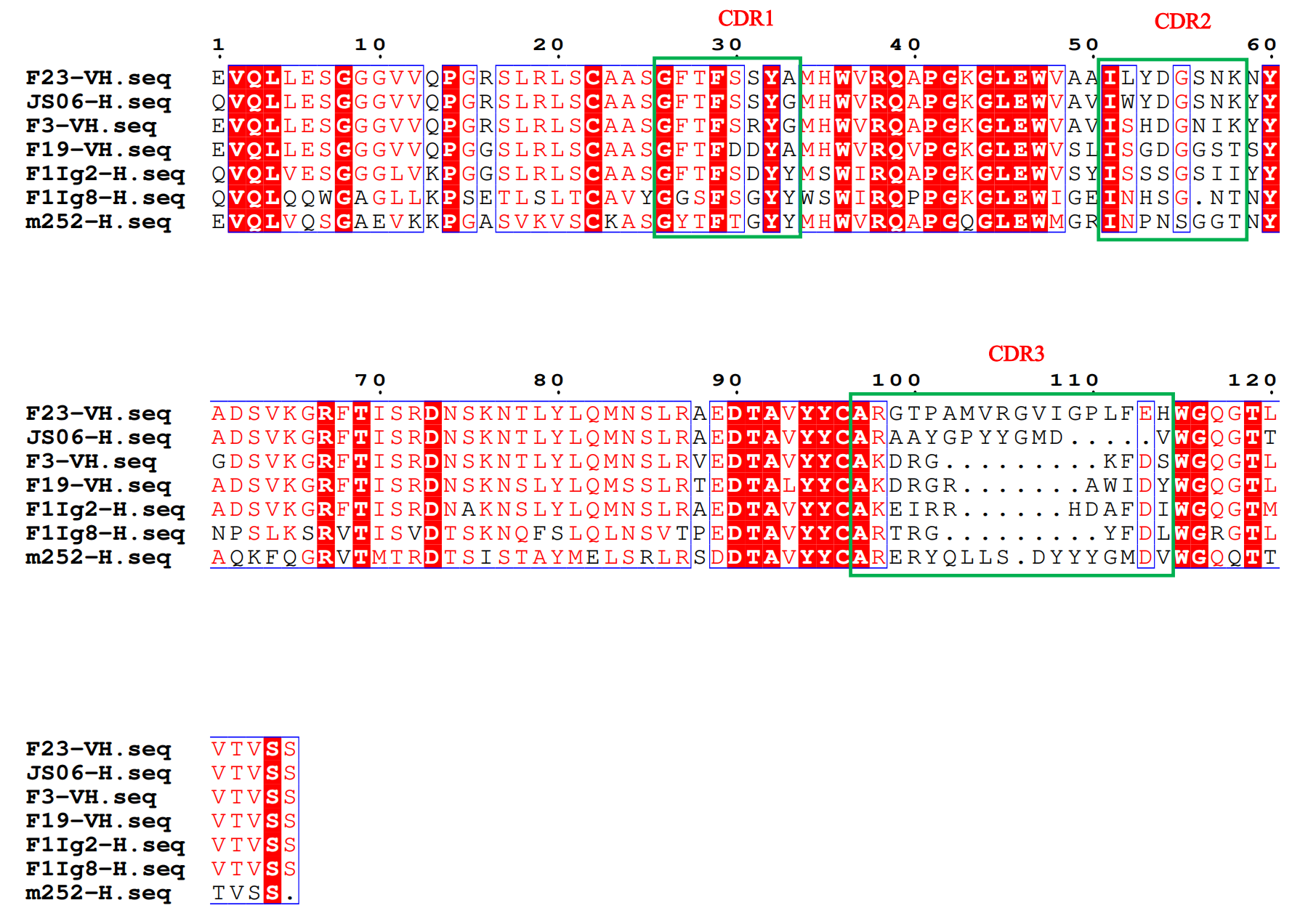


B


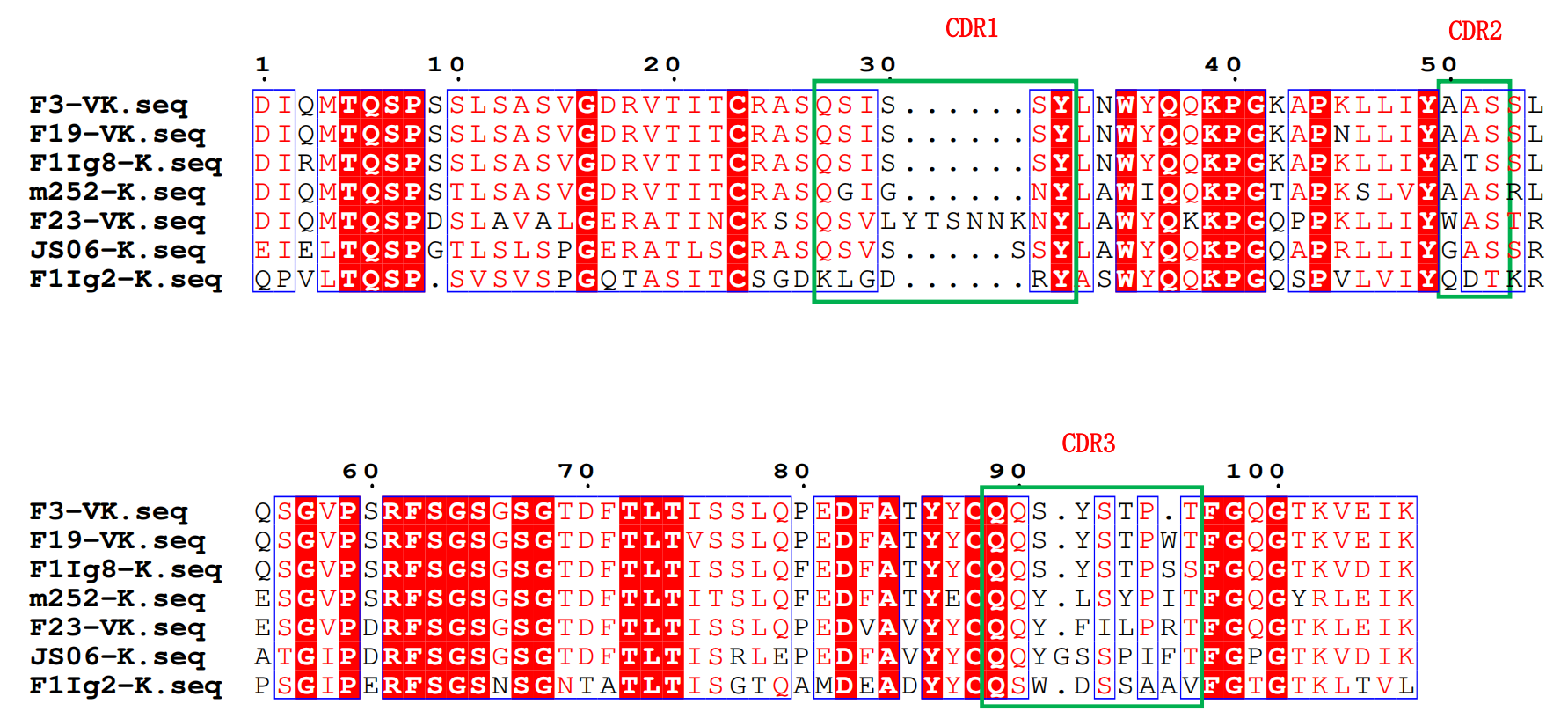


**FIG S3** **The amino acid sequence alignment of 7 mAbs.**

The variable regions of heavy chains (A) and light chains (B) of three human mAbs, αF1Ig2, αF1Ig8 and JS06.The amino acid sequences were aligned using the web servers of Clustal Omega (<https://www.ebi.ac.uk/Tools/msa/clustalo>) and ESPript 3.0 (<http://espript.ibcp.fr/ESPript/ESPript>). CDRs of heavy chains were highlighted by green-bordered boxes.


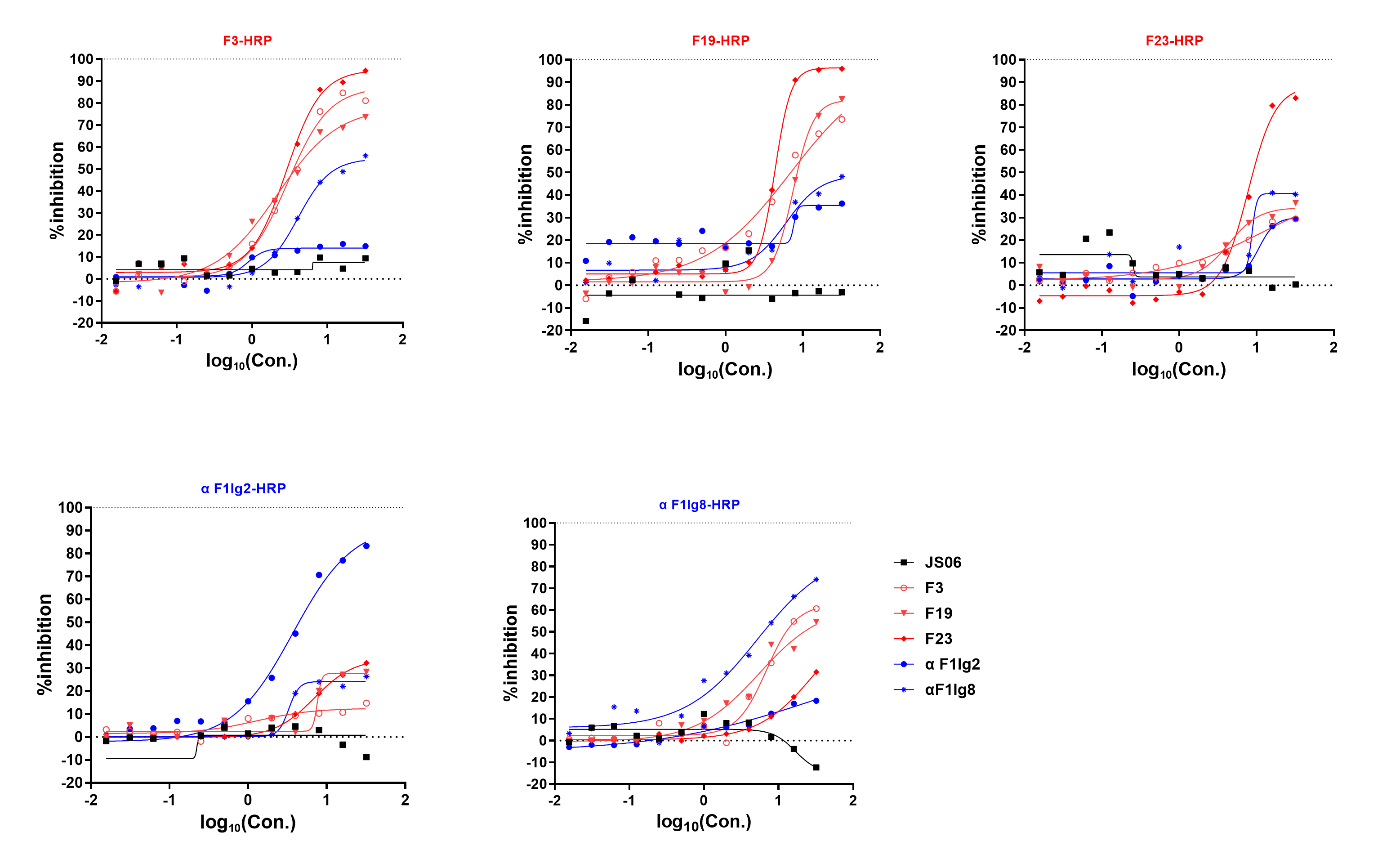


**FIG S4 Competition binding curves of mAbs by ELISA.**

F3, F19, and F23 were in red, and αF1Ig2 and αF1Ig8 were in blue. A summary of percent competitive binding is shown in Figure 4.

**TABLE S1 Panning results of the human ScFv library.**

| Round of panning | Input (PFU/ml) | Output (PFU/ml) | Output/Input | Enrichment | |
| --- | --- | --- | --- | --- | --- |
|  |  |  |  | Per round | Total |
| 1 | 5×10^11^ | 2×10^5^ | 4×10^-7^ | - | - |
| 2 | 5×10^11^ | 7×10^5^ | 1.4×10^-6^ | 3.5× | 3.5× |
| 3 | 5×10^11^ | 2×10^7^ | 4×10^-5^ | 28.6× | 100× |

The library was panned by F1 antigen for 3 rounds with increasing washing stringency and decreasing F1 concentration. Input and output titers were evaluated throughout 3 selection rounds. The enrichment of each round and total enrichment were calculated. The recovery of phages increased about 100 times after three rounds of panning, indicating that the enrichment ratio was within the normal level.

**TABLE S2 The gene characteristics of F3, F19 and F23.**

| Ab clone | V gene | Somatic mutation rate in V gene (% nucleotide) | D gene | J gene | Somatic mutation rate in V gene (%) | Amino acid No. Of CDR |
| --- | --- | --- | --- | --- | --- | --- |
| F3 | IGHV3-30*18 | 3.82 | IGHD3-10*0 | IGHJ4*02 | 10.64 | 8.8.9 |
|  | IGKV1-39*01 | 0.36 | - | IGKJ1*01 | 0.00 | 8.3.8 |
| F19 | IGHV3-43*02 | 3.12 | IGHD1-1*01 | IGHJ4*02 | 8.51 | 8.8.11 |
|  | IGKV1-39*01 | 1.08 | - | IGKJ1*01 | 2.78 | 8.3.8 |
| F23 | IGHV3-30*04 | 2.43 | IGHD3-10*01 | IGHJ4*02 | 12.77 | 8.8.18 |
|  | IGKV4-1*03 | 4.38 | - | IGKJ2*01 | 8.11 | 12.3.9 |
| m252 | IGHV1-2*02 | 17.36 | IGHD2-2*01 | IGHJ6*02 | 38.98 | 8.8.17 |
|  | IGKV1-16*01 | 26.52 |  | IGKJ2*01 | 24.32 | 6.3.9 |
| αF1Ig2 | IGHV3-11*04 | 25.35 | IGHD1-14*01 | IGHJ3*02 | 24.49 | 8.8.12 |
|  | IGLV3-1*01 | 20.07 |  | IGLJ6*01 | 20.59 | 6.3.9 |
| αF1Ig8 | IGHV4-34*07 | 21.83 | IGHD3-10*01 | IGHJ4*02 | 27.66 | 8.7.9 |
|  | IGKV1-39*01 | 28.31 |  | IGKJ1*01 | 25 | 6.3.9 |

**Note:** The V, D, and J sequences were compared by using the IMGT database. ‘-’ denotes the gene without the segment.

**TABLE S3 Amino acid sequences of the peptides used in ELISA.**

| NO. | AA sequence | NO. | AA sequence |
| --- | --- | --- | --- |
| P1 | MKKISSVIAIALFGTIA | P12 | AAGDPMYLTFTSQDGNNH |
| P2 | IAIALFGTIATANAADL | P13 | TFTSQDGNNHQFTTKVI |
| P3 | TIATANAADLTASTTATA | P14 | NNHQFTTKVIGKDSRDF |
| P4 | DLTASTTATATLVEPARI | P15 | KVIGKDSRDFDISPKV |
| P5 | TATLVEPARITLTYKEGA | P16 | SRDFDISPKVNGENLV |
| P6 | RITLTYKEGAPITMDNGNI | P17 | SPKVNGENLVGDDVVLA |
| P7 | APITMDNGNIDTELLV | P18 | NLVGDDVVLATGSQDFFV |
| P8 | NGNIDTELLVGTLTLGGY | P19 | LATGSQDFFVRSIGSK |
| P9 | LVGTLTLGGYKTGTTSTSV | P20 | DFFVRSIGSKGGKLAAGK |
| P10 | YKTGTTSTSVNFTDAA | P21 | SKGGKLAAGKYTDAVTV |
| P11 | STSVNFTDAAGDPMYLTF | P22 | AGKYTDAVTVTVSNQ |

**TABLE S4 Protection of Balb/c mice by mAbs against challenges of *Y. pestis* 141 strain.**

| mAb | Amount Given (µg) | Challenge Dose (MLD) | No. Survivors (after 20 Days) | Mean Time to Death (Days) |
| --- | --- | --- | --- | --- |
| F3 | 500 | 100 | 2/2 | >20 |
| F3 | 100 | 100 | 2/2 | >20 |
| F19 | 500 | 100 | 2/2 | >20 |
| F19 | 100 | 100 | 2/2 | >20 |
| F23 | 500 | 100 | 2/2 | >20 |
| F23 | 100 | 100 | 2/2 | >20 |
| Mix ^a^ | 500 | 100 | 2/2 | >20 |
| Mix | 100 | 100 | 2/2 | >20 |
| 100MLD^b^ | - | 100 | 0/8 | 4.875 |

^a^ Mix means F3, F19 and F23 mixed in equal amounts. B ^b^ 100MLD means control mice only received 100 MLD of *Y. pestis* 141 strain.
